# Dataset on concentration and enrichment factor of rare earth elements (REEs) in sediments of Linggi River, Malaysia

**DOI:** 10.1016/j.dib.2019.103983

**Published:** 2019-05-24

**Authors:** Md Suhaimi Elias, Shariff Ibrahim, Kamarudin Samuding, Nesamalar Kantasamy, Jeremy Andy Dominic Daung, Shamsiah Ab Rahman, Azian Hashim

**Affiliations:** a School of Chemistry and Environment, Faculty of Applied Sciences, Universiti Teknologi MARA (UiTM), 40450, Shah Alam, Selangor, Malaysia; b Analytical Chemistry Application Group (ACA), Waste and Environmental Technology Division, Malaysian Nuclear Agency, Bangi, 43000, Kajang, Selangor, Malaysia; c Environmental Tracer Application Group (E-TAG), Waste and Environmental Technology Division, Malaysian Nuclear Agency, Bangi, 43000, Kajang, Selangor, Malaysia

**Keywords:** Rare earth elements, Enrichment factor, Sediment, Linggi river

## Abstract

This study is on the distribution of rare earth elements (REEs) concentrations in sediments collected from 113 sampling locations of Linggi River. The analysis of sediment samples was performed by Neutron Activation Analysis (NAA) and Inductively Coupled Plasma – Mass spectrometer (ICP-MS). The main compositions of Linggi river sediments were silt > sand > clay. The mean of total concentrations of REEs (ΣREE), light REEs (ΣLREE) and heavy REEs (ΣHREE) in Linggi sediment were 249, 228, and 22.0 mg/kg, respectively. The results of Linggi river sediment were normalised to several reference shale values. REEs of Linggi river sediments were comparable to MUQ reference shale values. Enrichment factors (EF) of mean values indicate Linggi River sediment can be categorised as having minor to moderate enrichment.

**Table:** Specifications table

Subject area	Environmental Sciences
More specific subject area	Rare earth elements (REEs) pollution in sediment of Linggi River
Type of data	Tables and figures
How data was acquired	Neutron Activation Analysis (NAA) and Inductively Coupled Plasma – Mass Spectrometry (ICP-MS) -Model Elan 6000, Perkin Elmer.
Data format	Raw and analysed
Experimental factors	Linggi River sediment samples compared to several reference shale values. The enrichment factor (EF) was applied to identify the enrichment of REEs in Linggi sediment and possible sources of pollution.
Experimental features	Determination of REEs such as La, Ce, Pr, Nd, Sm, Eu, Gd, Tb, Ho, Er, Tm, Yb and Lu concentrations.
Data source location	Linggi River sediment of Negeri Sembilan, Malaysia
Data accessibility	Data is in this article
Related research article	B.S. Kamber, A. Greig, K.D. Collerson. 2005. A new estimate for the composition of weathered young upper continental crust from alluvial sediments, Queensland, Australia, Geochim. Cosmochim. Acta. 69, 1041–1058. https://doi.org/10.1016/j.gca.2004.08.020.

**Table:** 

**Value of the Data** • The dataset is presented on the concentrations of rare earth element (REEs) in the sediments of Linggi River which can serve as a baseline for future references. • Normalization of Linggi sediment to several reference shale values showed Linggi sediment samples are comparable to MUQ reference shale value. • This data is useful to identify the major REEs pollution in Linggi River sediments.

## 1 Data

**Fig. 1:**
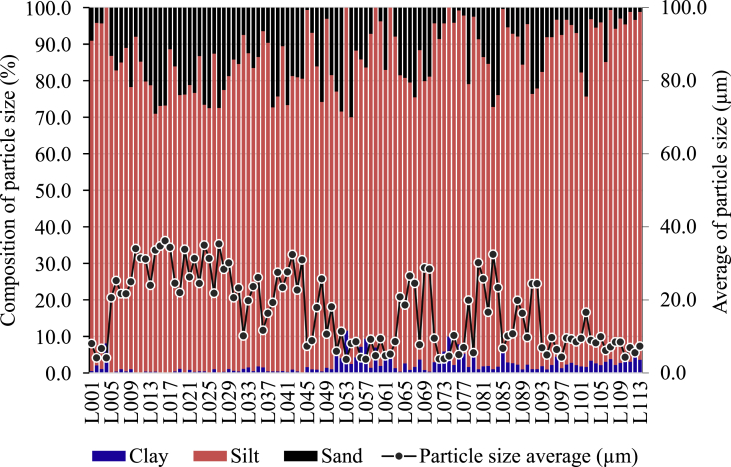
Composition and average of particle size of Linggi River sediment.

**Fig. 2:**
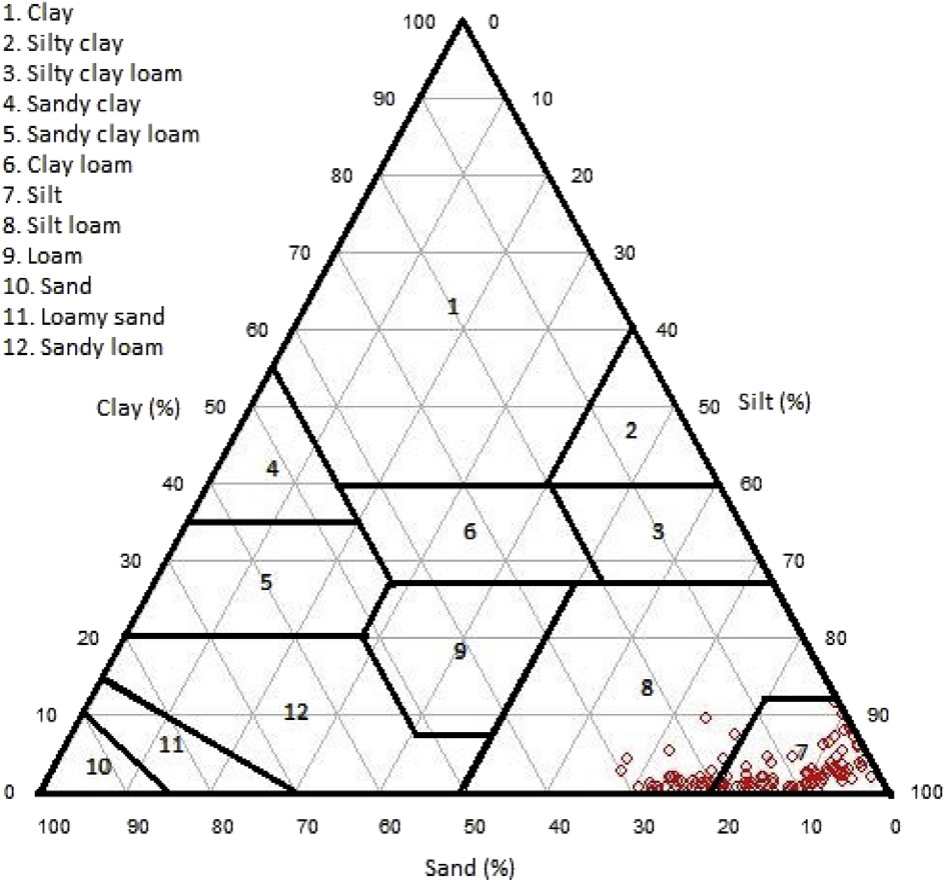
Plotting results of Linggi River sediment textural classification.

Composition and average of particle size of Linggi River sediment are depicted in Fig. 1. Major compositions of Linggi River sediment are silt > sand > clay. The average of particle size of Linggi River sediment was less than 35 μm in all sampling locations (Fig. 1). High content of clay and silt (particle size average < 63 μm) in sediment is adequate for analysis of elemental content including rare earth elements concentration. Sediment chart and plotting results of textural classification of the Linggi River sediment is depicted in Fig. 2. The texture of Linggi River sediment can be classified as slit and silt loam (Fig. 2).

Light rare earth elements (LREEs) consist of La, Ce, Pr, Nd, Sm and Eu whereas heavy rare earth elements (HREEs) consist of Gd, Tb, Ho, Er, Tm, Yb, and Lu. The results obtained for the statistical summary of the minimum (min), maximum (max), mean, total concentration of each element, ΣREE, ΣLREE, and ΣHREE of sediment samples that were collected from 113 locations of Linggi River are tabulated in Table 1. Generally, LREEs were the major concentrations contributing to ΣREE in Linggi River sediments. The ΣLREE in Linggi River sediments were higher than ΣHREE, indicating the LREEs in the sediment may have originated from terrigenous and riverine sources. The analytical results of standard reference material (SRM) and experimental values of IAEA SL-1 and BHVO-1 are tabulated in Table 1. The relative bias (%) values of REEs range from −18.9 to 14.2%. The relative bias of the REEs are within the range of the acceptable values (<±20%) [1], [2].

**Table 1:** The elemental concentrations (mg/kg), LREE, HREE, total REE, and statistical summary of REEs in the sediments of Linggi River.

Location	La	Ce	Pr	Nd	Sm	Eu	Gd	Tb	Ho	Er	Tm	Yb	Lu	LREE	HREE	Total REE
L001	37.1	115	11.6	31.7	7.47	0.93	5.21	0.85	1.03	1.79	0.45	3.61	0.54	204	13.5	218
L002	20.3	88.2	10.0	21.9	4.31	0.52	4.29	0.53	0.64	1.08	0.26	2.59	0.36	145	9.74	155
L003	74.1	161	12.9	65.3	12.7	0.75	7.86	1.25	0.69	1.82	0.36	5.06	0.71	327	17.7	344
L004	21.5	64.7	3.79	31.9	4.01	0.57	2.36	0.16	0.27	0.77	0.17	1.57	0.23	126	5.53	132
L005	72.9	138	19.9	51.3	13.8	0.73	12.7	1.52	1.00	2.54	0.46	5.69	0.83	297	24.7	321
L006	158	171	30.9	110	39.5	1.17	22.2	4.37	2.35	3.60	0.69	14.6	1.92	511	49.7	561
L007	69.0	136	28.4	58.8	18.0	2.21	16.2	2.70	5.79	10.6	2.82	11.3	1.60	312	50.9	363
L008	56.2	131	17.6	45.0	14.9	1.55	9.94	2.25	3.37	6.14	1.67	9.92	1.45	266	34.7	301
L009	56.8	109	21.5	61.6	14.3	1.35	11.9	2.02	3.98	7.24	1.93	9.48	1.33	265	37.8	303
L010	45.8	112	15.3	53.6	10.2	0.90	6.74	1.28	1.53	2.76	0.71	5.61	0.84	238	19.5	258
L011	53.7	112	19.2	61.4	12.9	1.00	8.00	1.97	1.95	3.59	0.95	11.2	1.62	260	29.3	289
L012	44.1	98.5	16.4	54.7	11.6	1.04	8.16	1.72	2.29	4.16	1.12	8.71	1.27	226	27.4	254
L013	44.6	117	18.1	56.5	12.1	1.33	9.36	1.93	2.80	4.89	1.36	9.17	1.30	249	30.8	280
L014	58.3	124	18.9	81.5	15.1	1.03	6.96	2.21	1.89	3.47	0.95	11.8	1.72	299	29.0	328
L015	43.7	94.9	11.0	62.1	11.6	1.06	8.93	1.53	1.66	4.98	0.79	6.98	1.10	224	26.0	250
L016	46.0	106	10.6	53.6	12.0	1.24	9.07	1.71	1.74	5.11	0.84	8.01	1.08	229	27.6	257
L017	45.2	100	12.8	41.6	12.1	0.92	9.59	1.53	1.55	4.59	0.76	9.09	1.16	212	28.3	241
L018	44.0	113	9.35	36.6	12.9	1.41	8.30	1.67	1.64	4.77	0.81	8.26	1.09	217	26.5	244
L019	41.0	102	8.93	26.5	11.0	1.10	7.72	1.36	1.49	4.29	0.72	6.98	0.83	191	23.4	214
L020	42.9	100	7.63	29.1	11.4	1.11	6.53	1.50	1.29	3.77	0.64	8.37	1.13	193	23.2	216
L021	41.8	97.0	9.58	25.2	11.6	1.12	9.18	1.35	1.90	5.59	1.00	6.89	0.86	186	26.8	213
L022	47.6	117	13.0	38.2	12.6	1.38	11.3	1.51	2.18	6.40	1.13	8.40	0.99	230	31.9	262
L023	38.2	87.7	8.23	25.6	9.98	0.95	6.34	1.29	1.14	3.36	0.56	7.59	0.91	171	21.2	192
L024	47.6	105	10.4	59.5	12.1	1.00	8.76	1.77	1.71	5.00	0.83	7.63	1.14	235	26.8	262
L025	40.2	91.2	9.81	27.2	10.8	0.98	7.79	1.49	1.41	4.15	0.69	8.08	1.06	180	24.7	205
L026	44.4	108	11.2	27.5	12.4	1.22	9.62	1.54	1.90	5.51	0.92	8.35	1.02	205	28.8	234
L027	44.1	102	13.0	33.4	11.7	1.02	9.95	1.45	1.67	4.94	0.81	8.09	0.95	206	27.9	233
L028	43.6	100	9.96	50.9	11.1	1.03	7.86	1.46	1.42	4.21	0.69	6.99	1.02	217	23.7	240
L029	36.9	114	7.35	35.1	8.83	1.44	5.07	1.31	1.12	2.76	0.55	7.72	1.11	204	19.6	224
L030	44.0	86.4	10.6	35.9	10.4	1.58	7.11	1.94	1.60	3.91	0.77	5.58	0.87	189	21.8	211
L031	41.0	102	9.47	60.9	10.0	0.94	7.72	1.19	1.37	4.04	0.65	5.05	0.72	224	20.8	245
L032	45.6	124	9.86	29.4	9.68	1.23	6.35	1.09	1.32	3.22	0.62	5.41	0.61	220	18.6	239
L033	49.0	100	7.63	33.0	8.04	1.02	4.17	0.84	0.76	1.85	0.34	3.32	0.56	199	11.9	211
L034	29.4	62.4	8.06	19.4	6.29	0.78	4.81	0.68	0.86	2.12	0.41	3.94	0.67	126	13.5	140
L035	57.2	66.1	12.0	38.9	9.16	1.19	6.53	1.04	1.24	3.00	0.57	4.87	0.67	185	17.9	202
L036	40.4	92.1	10.5	18.2	8.64	1.02	7.24	0.97	1.10	3.12	0.51	4.91	0.65	171	18.5	189
L037	45.4	95.2	10.6	25.8	10.9	1.22	8.80	1.17	1.50	4.30	0.71	6.04	0.64	189	23.2	212
L038	45.2	101	10.4	27.4	9.43	1.24	7.65	1.06	1.15	3.32	0.54	5.25	0.66	194	19.6	214
L039	39.6	99.2	10.8	30.5	8.61	1.14	7.57	1.25	1.23	3.57	0.59	5.49	0.83	190	20.5	210
L040	42.2	90.8	10.9	34.8	9.14	1.17	8.00	1.03	1.30	3.74	0.62	4.94	0.60	189	20.2	209
L041	40.7	79.9	8.16	38.8	8.70	1.08	6.35	1.20	1.03	2.98	0.49	5.27	0.72	177	18.0	195
L042	52.5	99.4	12.5	34.6	9.50	1.26	6.33	1.18	1.33	2.45	0.59	4.74	0.66	210	17.3	227
L043	49.7	103	12.8	42.3	10.8	1.35	7.66	1.46	1.65	3.07	0.77	5.99	0.80	220	21.4	241
L044	39.7	77.0	9.83	34.3	8.52	1.06	6.90	1.11	1.06	2.02	0.50	4.97	0.68	170	17.2	188
L045	46.5	92.4	9.92	27.4	9.86	1.26	5.45	1.25	1.31	2.49	0.62	5.54	0.78	187	17.4	205
L046	42.1	89.7	10.7	39.5	9.98	1.21	6.14	1.39	1.49	2.80	0.70	5.53	0.72	193	18.8	212
L047	40.4	89.6	8.56	32.2	9.24	1.32	4.90	1.33	1.23	2.35	0.59	6.10	0.84	181	17.3	199
L048	43.1	103	14.9	39.8	10.5	1.43	6.75	1.43	1.87	3.35	0.90	6.49	0.84	212	21.6	234
L049	38.9	83.3	10.2	38.0	8.97	1.17	4.68	1.27	1.31	2.36	0.64	6.01	0.79	181	17.1	198
L050	51.7	113	9.91	39.1	11.9	1.57	4.72	1.63	1.33	2.40	0.65	7.45	1.02	227	19.2	246
L051	77.6	169	15.9	70.4	15.3	1.20	10.9	1.77	1.45	4.20	0.66	9.90	1.13	349	30.1	379
L052	101	177	14.7	85.1	17.9	1.04	9.50	1.58	1.18	3.33	0.56	8.15	0.80	397	25.1	422
L053	47.8	118	9.43	38.1	9.79	1.25	7.83	1.21	1.48	4.31	0.71	6.85	0.73	224	23.1	247
L054	33.7	80.9	5.15	41.5	7.21	1.02	3.87	0.80	0.69	2.00	0.33	5.27	0.56	169	13.5	183
L055	46.4	114	7.84	56.3	9.22	1.35	6.54	1.13	1.16	3.49	0.57	7.36	0.75	236	21.0	257
L056	44.4	119	7.72	28.1	8.86	0.98	6.35	0.89	1.16	3.46	0.57	4.44	0.74	209	17.6	227
L057	43.7	114	8.01	37.7	9.06	1.06	6.64	1.15	1.24	3.70	0.61	5.40	1.06	214	19.8	233
L058	57.7	148	3.72	51.2	10.3	2.30	3.98	1.28	0.87	2.59	0.42	4.78	0.82	273	14.7	288
L059	52.4	138	7.73	38.2	10.2	1.16	7.26	1.18	1.55	4.85	0.77	4.89	0.90	248	21.4	269
L060	55.5	127	3.43	36.8	9.47	1.77	3.00	1.12	0.57	1.72	0.28	4.92	0.90	234	12.5	247
L061	46.7	132	11.1	52.6	11.9	0.66	8.27	1.74	1.23	3.61	0.58	6.70	1.06	255	23.2	278
L062	55.2	153	12.6	77.3	15.9	1.06	9.55	1.71	1.54	4.44	0.69	4.84	0.88	315	23.7	338
L063	42.9	133	10.1	49.6	11.3	0.84	7.38	1.45	1.15	3.33	0.53	5.33	0.93	247	20.1	267
L064	36.9	107	11.5	42.5	9.46	0.80	7.22	1.05	1.72	4.20	0.51	4.25	0.72	208	19.7	227
L065	32.5	100	10.6	37.6	9.17	0.60	8.00	1.49	1.60	3.90	0.50	4.91	0.94	190	21.3	211
L066	34.7	91.9	13.3	33.1	7.84	0.58	8.55	1.40	1.20	3.45	0.57	5.30	0.89	181	21.4	203
L067	50.6	134	11.4	51.0	11.0	0.78	8.98	1.56	1.29	3.94	0.62	5.88	0.99	258	23.3	282
L068	47.1	124	13.6	40.4	9.64	0.84	11.1	1.20	1.62	4.71	0.72	3.75	0.76	235	23.9	259
L069	33.2	89.2	15.0	37.3	7.17	0.54	12.4	1.15	1.78	5.49	0.88	4.33	0.79	182	26.9	209
L070	38.5	102	8.61	43.0	8.58	0.46	5.85	1.32	0.68	2.10	0.33	6.38	1.08	201	17.7	219
L071	43.8	106	11.1	41.7	9.06	1.35	8.48	1.39	1.34	4.05	0.63	4.83	0.79	213	21.5	235
L072	43.6	115	11.3	38.8	9.27	1.36	8.84	1.53	1.43	4.35	0.67	4.99	0.89	219	22.7	242
L073	46.9	123	12.5	40.0	9.92	1.27	8.39	1.53	1.91	4.37	0.90	5.02	0.86	234	23.0	257
L074	47.8	129	13.5	44.9	10.3	1.32	9.05	1.51	2.10	4.82	0.99	4.01	0.89	247	23.4	270
L075	47.0	119	13.5	42.3	9.73	1.22	8.77	1.26	2.06	4.74	0.98	4.76	0.78	232	23.3	256
L076	51.3	129	16.5	36.6	10.0	1.86	9.59	1.42	1.96	4.48	0.92	5.50	0.91	245	24.8	270
L077	44.1	111	14.1	34.7	9.27	1.09	9.03	1.31	2.13	4.89	1.01	4.28	0.74	214	23.4	237
L078	39.4	103	8.11	41.1	8.24	1.36	5.81	1.31	0.90	2.68	0.42	4.53	0.87	201	16.5	217
L079	43.9	107	9.50	30.5	8.82	1.23	7.18	1.23	1.19	3.54	0.56	4.91	0.78	200	19.4	220
L080	37.2	84.8	10.1	27.2	8.02	1.24	6.87	1.29	0.87	2.63	0.41	4.80	0.84	169	17.7	186
L081	53.1	147	10.3	53.3	10.6	1.45	6.81	1.77	0.91	2.51	0.43	8.37	1.23	276	22.0	298
L082	52.6	130	10.2	45.1	10.5	1.59	7.21	1.62	1.17	3.20	0.56	7.27	0.95	250	22.0	272
L083	35.4	96.2	5.92	38.9	7.72	0.90	5.06	1.38	0.56	1.70	0.27	7.40	1.16	185	17.5	203
L084	41.4	119	7.55	48.1	8.15	1.31	6.17	1.46	0.71	2.18	0.34	7.70	1.09	225	19.6	245
L085	51.9	144	9.08	41.7	9.50	1.64	8.16	1.39	1.01	3.08	0.48	5.64	0.48	258	20.2	278
L086	54.3	140	6.21	50.6	9.94	2.18	7.11	1.41	0.97	2.93	0.46	6.67	0.93	263	20.5	284
L087	48.0	123	4.60	46.9	8.92	1.79	5.89	1.31	0.71	2.18	0.34	5.33	0.55	233	16.3	249
L088	45.4	120	4.67	60.0	9.16	1.13	5.12	1.15	0.56	1.69	0.26	5.79	0.84	241	15.4	256
L089	41.2	106	10.9	36.5	7.44	1.12	7.73	1.00	1.04	3.20	0.50	5.23	0.79	203	19.5	223
L090	51.3	131	10.9	41.8	8.73	1.62	7.85	1.32	1.11	3.37	0.51	5.09	0.79	245	20.0	265
L091	70.8	140	17.2	77.4	13.8	1.63	12.1	3.42	1.31	4.05	0.63	10.5	1.44	321	33.4	354
L092	50.2	140	9.38	47.8	10.3	1.51	7.31	1.53	1.11	3.35	0.54	7.51	1.03	260	22.4	282
L093	49.8	135	9.11	43.7	9.84	1.33	6.82	1.62	0.93	2.82	0.44	7.42	1.10	249	21.2	270
L094	45.4	130	8.88	46.4	9.01	1.55	6.88	1.39	1.06	3.17	0.50	6.10	0.74	241	19.8	261
L095	49.5	128	11.5	35.2	9.89	1.87	9.74	1.21	1.70	5.09	0.82	6.30	0.53	236	25.4	262
L096	78.3	147	10.8	78.2	15.5	1.79	8.60	3.24	1.73	5.00	0.75	11.8	1.58	332	32.7	365
L097	47.1	124	9.97	37.7	9.14	1.76	7.93	1.04	1.30	3.89	0.61	5.46	0.44	230	20.7	251
L098	54.8	138	10.4	42.9	11.3	1.84	9.37	1.51	1.77	5.32	0.85	7.17	0.61	259	26.6	286
L099	51.1	130	10.0	40.5	9.09	1.87	8.14	1.05	1.29	3.92	0.60	5.03	0.40	242	20.4	263
L100	53.7	131	10.1	37.0	10.3	1.74	8.18	1.08	1.29	3.96	0.61	5.71	0.53	244	21.4	265
L101	48.4	124	8.74	38.4	8.44	1.68	6.69	0.98	0.94	2.83	0.44	5.66	0.70	229	18.2	247
L102	53.5	142	9.84	39.5	10.9	1.53	7.81	1.31	1.08	3.22	0.50	7.89	1.27	258	23.1	281
L103	53.4	135	10.0	46.5	10.6	1.59	8.11	1.21	1.25	3.78	0.59	5.05	0.50	257	20.5	277
L104	48.6	134	9.89	36.2	9.91	1.78	8.41	1.11	1.32	3.96	0.61	6.15	0.54	240	22.1	262
L105	42.2	100	8.28	34.8	8.47	1.49	6.83	0.95	1.06	3.18	0.49	4.71	0.60	195	17.8	213
L106	46.8	108	9.21	28.4	9.51	1.71	7.90	0.52	1.28	3.88	0.60	5.17	0.46	203	19.8	223
L107	50.1	133	9.81	36.5	10.2	1.71	7.98	1.17	1.28	3.83	0.61	5.50	0.65	241	21.0	262
L108	47.6	120	8.25	39.4	9.25	1.78	6.58	1.04	0.99	2.99	0.46	4.99	0.70	227	17.8	244
L109	48.2	127	9.48	37.1	9.31	1.85	7.55	0.99	1.09	3.30	0.51	5.02	0.72	233	19.2	252
L110	42.4	92.7	8.41	40.5	6.89	1.25	7.02	1.15	1.07	3.24	0.50	4.46	0.57	192	18.0	210
L111	52.2	76.5	8.53	34.9	8.57	0.98	7.07	1.09	1.01	3.05	0.47	4.08	0.58	182	17.4	199
L112	46.4	93.6	8.52	37.6	7.69	0.84	7.31	0.97	1.07	3.23	0.50	4.02	0.58	195	17.7	212
L113	48.2	102	8.14	32.7	8.21	1.43	6.82	1.04	1.03	3.07	0.47	4.14	0.56	200	17.1	218
N	113	113	113	113	113	113	113	113	113	113	113	113	113	113	113	113
Min	20.3	62.4	3.4	18.2	4.01	0.46	2.36	0.16	0.27	0.77	0.17	1.57	0.23	126	5.5	132
Max	158	177	30.9	110	39.5	2.30	22.2	4.37	5.79	10.6	2.82	14.6	1.92	511	50.9	561
Sum	5461	12849	1242	4836	1180	142	877	157	158	410	74.6	706	97.9	25709	2481	28191
Mean	48.3	114	11.0	42.8	10.4	1.26	7.76	1.39	1.40	3.63	0.66	6.25	0.87	228	22.0	249
Standard error	1.40	2.10	0.39	1.36	0.34	0.04	0.23	0.05	0.06	0.12	0.03	0.19	0.03	4.79	0.59	5.19
Variance	221	499	17.2	209	12.9	0.14	5.99	0.27	0.45	1.69	0.11	4.25	0.09	2593	40.0	3039
Standard deviation	14.9	22.3	4.14	14.5	3.59	0.37	2.45	0.52	0.67	1.30	0.33	2.06	0.29	50.9	6.3	55.1
Median	46.4	112	10.1	39.1	9.9	1.23	7.66	1.31	1.29	3.47	0.60	5.58	0.83	224	21.0	245
25 percentile	42.2	99.3	8.7	34.8	8.9	1.02	6.56	1.13	1.06	2.83	0.50	4.92	0.67	194	18.0	213
75 percentile	51.5	130	12.5	48.9	11.3	1.52	8.77	1.53	1.63	4.30	0.76	7.44	1.03	247	23.8	269
Coefficient of variation	30.8	19.6	37.7	33.8	34.3	29.6	31.5	37.5	48.0	35.8	49.7	33.0	33.7	22.4	28.8	22.1
Detection limit	0.001	0.50	0.05	0.01	0.005	0.001	0.02	0.001	0.001	0.001	0.001	0.001	0.001	–	–	–
SRM Certificate value	52.6^#^	117^#^	18*	43.8^#^	9.25^#^	1.60^#^	6.40*	1.40^#^	0.99*	2.42*	0.33*	3.42^#^	0.54^#^	–	–	–
SRM Analysis value	45.9	99.1	14.6	42.6	10.1	1.67	7.31	1.26	1.11	2.66	0.37	3.41	0.45	–	–	–
Relative bias (%)	−12.7	−15.3	−18.9	−2.75	9.19	4.70	14.2	−10.1	11.7	9.77	11.0	−0.39	−15.8	–	–	–

Standard reference materials (SRM) values were obtained from IAEA SL-1 (#) and BHVO-1 (*) certificate.

**Fig. 3:**
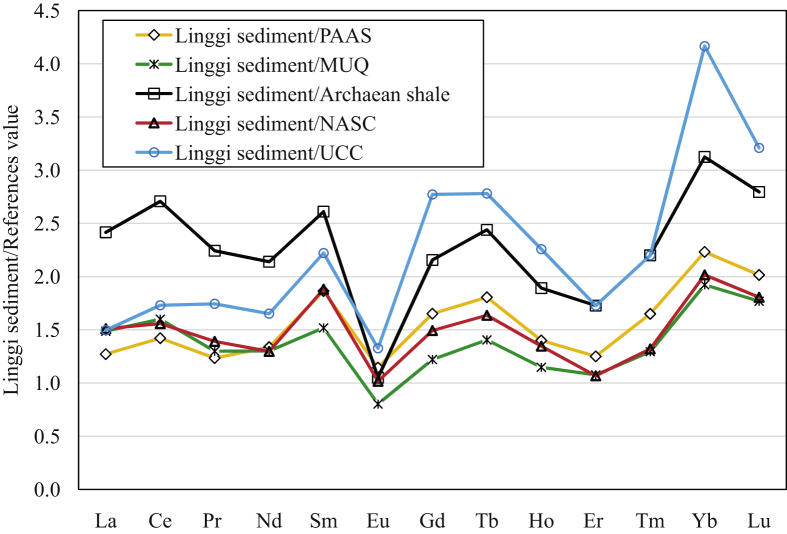
Normalization of Linggi sediment to other reference values of REEs.

REEs in Linggi sediments are normalised to several reference shale values such as post-Archaean Australian Shale (PAAS), mud from Queensland (MUQ), Archaean shale, North American Shale Composite (NASC) and upper continental crust (UCC) as shown in Fig. 3. The data of reference shale and Linggi sediment values of REEs and the ratio of Linggi sediment to other reference values are tabulated in Table 2. The REEs data from Linggi sediment display almost a flat pattern normalised to MUQ reference shale values, with REEs values of 0.8–1.60 except for Yb and Lu (Fig. 3). This indicate the Linggi sediment are comparable to the MUQ reference values due to not much fluctuation of REEs compared to the other reference values such as PAAS, Archaean shale, NASC and UCC.

**Table 2:** Linggi sediment, reference values (MUQ, PAAS, Archaean shale, NASC and UCC) of REEs and normalization ratios of Linggi sediment to reference values REEs.

	La	Ce	Pr	Nd	Sm	Eu	Gd	Tb	Ho	Er	Tm	Yb	Lu
Linggi sediment	48.3	114	11.0	42.8	10.4	1.26	7.76	1.39	1.40	3.63	0.66	6.25	0.87
MUQ(a)	32.5	71.1	8.46	32.9	6.88	1.57	6.36	0.99	1.22	3.37	0.51	3.25	0.49
PAAS(b)	38.0	80.0	8.90	32.0	5.60	1.10	4.70	0.77	1.00	2.90	0.40	2.80	0.43
Archaean shale(b)	20.0	42.0	4.90	20.0	4.00	1.20	3.60	0.57	0.74	2.10	0.30	2.00	0.31
NASC(c)	32.0	73.0	7.90	33.0	5.55	1.24	5.20	0.85	1.04	3.40	0.50	3.10	0.48
UCC(d)	32.3	65.7	6.30	25.9	4.70	0.95	2.80	0.50	0.62	2.10	0.30	1.50	0.27
Linggi sediment/MUQ	1.49	1.60	1.30	1.30	1.52	0.80	1.22	1.40	1.15	1.08	1.29	1.92	1.77
Linggi sediment/PAAS	1.27	1.42	1.23	1.34	1.86	1.15	1.65	1.81	1.40	1.25	1.65	2.23	2.02
Linggi sediment/Archaean shale	2.42	2.71	2.24	2.14	2.61	1.05	2.16	2.44	1.89	1.73	2.20	3.12	2.80
Linggi sediment/NASC	1.51	1.56	1.39	1.30	1.88	1.02	1.49	1.64	1.35	1.07	1.32	2.02	1.81
Linggi sediment/UCC	1.50	1.73	1.74	1.65	2.22	1.33	2.77	2.78	2.26	1.73	2.20	4.17	3.21

(a )[3].

(b )[4].

(c )[5].

(d )[6].

**Fig. 4:**
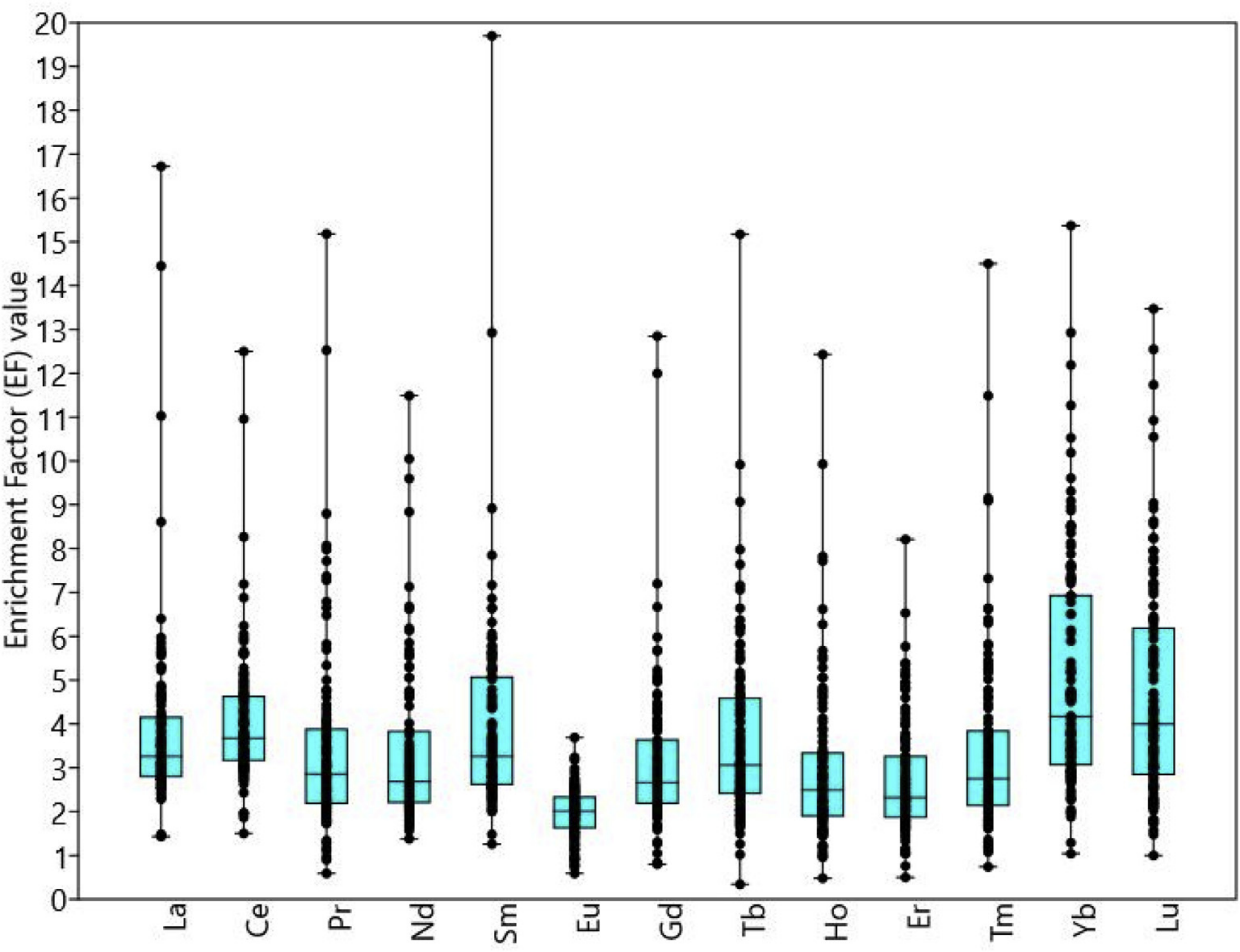
The EF value (jitter) and mean (box plot) of REEs in Linggi River sediment.

The enrichment factor (EF) is frequently used to evaluate the possible addition of an anthropogenic component and lithogenic processes [7], [8], [9], [10]. The calculation of the enrichment factor (EF) of Linggi sediment is according to Eq. (1).

(1) $$EF=\frac{\left(\frac{M_{analysis}}{Fe_{analysis}}\right)sample}{\left(\frac{M_{reference}}{Fe_{reference}}\right)MUQ}$$

where M_analysis._ is the concentration value of interest element in Linggi sediment sample, Fe_analysis_ is the concentration value of Fe in Linggi sediment sample, M_reference_ is the concentration value of interest element from reference shale value, (MUQ) and Fe_reference_ is the concentration value of Fe = 54300 mg/kg from MUQ [3]. EF can be categorised as follows: EF value ≤ 2.0, 2 to 3, 3 to 5, 5 to 10, 10 to 25, 25 to 50 and > 50 are no enrichment (shale), minor, moderate, moderately severe, severe, very severe and extreme enrichment, respectively [9], [10]. The EF values and summary statistics of REEs are tabulated in Table 3 whilst jitter and box plot of values and mean EF of REEs in Linggi river sediment are shown in Fig. 4. The 25 and 75 percentiles of EF values are shown as the lowest and highest of box plot of REEs, respectively. The values of 25 and 75 percentiles were 2.8, 3.2, 2.2, 2.2, 2.6, 1.6, 2.2, 2.4, 1.9, 1.9, 2.1, 3.1, 2.9) and 4.1, 4.6, 3.8, 3.8, 5.0, 2.3, 3.6, 4.5, 3.3, 3.3, 3.8, 6.9, 6.1 for La, Ce, Pr, Nd, Sm, Eu, Gd, Tb, Ho, Er, Tm, Yb and Lu, respectively. EF mean values of REEs ranged from 2.0 (Eu) to 5.1 (Yb) indicating Linggi River sediment can be categorised as having minor to moderate enrichment in most of the locations (Table 3).

**Table 3:** The EF values and statistical summary of REEs in Linggi River sediments.

Location	La	Ce	Pr	Nd	Sm	Eu	Gd	Tb	Ho	Er	Tm	Yb	Lu
L001	3.5	5.0	4.2	3.0	3.3	1.8	2.5	2.6	2.6	1.6	2.7	3.4	3.4
L002	1.5	2.9	2.8	1.6	1.5	0.8	1.6	1.3	1.2	0.8	1.2	1.9	1.7
L003	11.0	11.0	7.4	9.6	8.9	2.3	6.0	6.1	2.7	2.6	3.4	7.5	7.0
L004	1.4	2.0	1.0	2.1	1.3	0.8	0.8	0.3	0.5	0.5	0.7	1.0	1.0
L005	14.4	12.5	15.2	10.0	12.9	3.0	12.9	9.9	5.3	4.9	5.8	11.3	10.9
L006	16.7	8.3	12.5	11.5	19.7	2.5	12.0	15.2	6.6	3.7	4.6	15.4	13.5
L007	5.6	5.0	8.8	4.7	6.9	3.7	6.7	7.2	12.4	8.2	14.5	9.1	8.6
L008	4.8	5.1	5.8	3.8	6.1	2.8	4.4	6.4	7.7	5.1	9.1	8.5	8.3
L009	5.3	4.7	7.7	5.7	6.3	2.6	5.7	6.2	9.9	6.5	11.5	8.9	8.2
L010	5.3	6.0	6.8	6.1	5.6	2.2	4.0	4.9	4.7	3.1	5.2	6.5	6.4
L011	5.9	5.6	8.1	6.6	6.6	2.3	4.5	7.1	5.7	3.8	6.6	12.2	11.7
L012	4.5	4.6	6.5	5.6	5.6	2.2	4.3	5.8	6.3	4.1	7.3	9.0	8.6
L013	4.7	5.6	7.3	5.9	6.0	2.9	5.0	6.6	7.8	4.9	9.1	9.6	9.0
L014	6.4	6.2	8.0	8.8	7.9	2.3	3.9	8.0	5.5	3.7	6.6	12.9	12.5
L015	4.8	4.7	4.6	6.7	6.0	2.4	5.0	5.5	4.8	5.2	5.4	7.6	7.9
L016	4.6	4.9	4.1	5.3	5.7	2.6	4.7	5.6	4.6	4.9	5.4	8.0	7.2
L017	5.2	5.3	5.7	4.8	6.6	2.2	5.7	5.8	4.8	5.1	5.6	10.5	8.9
L018	4.1	4.8	3.3	3.3	5.6	2.7	3.9	5.1	4.0	4.3	4.8	7.6	6.7
L019	4.0	4.5	3.3	2.5	5.1	2.2	3.8	4.3	3.9	4.0	4.5	6.8	5.4
L020	4.0	4.3	2.8	2.7	5.1	2.2	3.1	4.6	3.2	3.4	3.8	7.9	7.1
L021	4.2	4.4	3.7	2.5	5.5	2.3	4.7	4.4	5.1	5.4	6.4	6.9	5.7
L022	4.1	4.7	4.3	3.3	5.2	2.5	5.0	4.3	5.1	5.4	6.3	7.3	5.7
L023	4.7	4.9	3.9	3.1	5.8	2.4	4.0	5.2	3.7	4.0	4.4	9.3	7.4
L024	4.5	4.6	3.8	5.6	5.5	2.0	4.3	5.5	4.4	4.6	5.0	7.3	7.2
L025	3.7	3.8	3.4	2.5	4.7	1.8	3.6	4.5	3.4	3.7	4.0	7.4	6.4
L026	4.0	4.5	3.9	2.5	5.3	2.3	4.5	4.6	4.6	4.8	5.3	7.6	6.2
L027	4.4	4.7	5.0	3.3	5.6	2.1	5.1	4.8	4.5	4.8	5.2	8.1	6.3
L028	3.8	4.0	3.4	4.4	4.6	1.9	3.5	4.2	3.3	3.6	3.9	6.1	5.9
L029	4.0	5.7	3.1	3.7	4.5	3.2	2.8	4.7	3.2	2.9	3.8	8.4	8.0
L030	4.3	3.8	4.0	3.4	4.8	3.2	3.5	6.2	4.1	3.7	4.8	5.4	5.6
L031	3.2	3.7	2.9	4.7	3.7	1.5	3.1	3.1	2.9	3.1	3.3	4.0	3.8
L032	2.6	3.3	2.2	1.7	2.6	1.5	1.9	2.0	2.0	1.8	2.3	3.1	2.3
L033	2.9	2.7	1.7	1.9	2.3	1.3	1.3	1.6	1.2	1.1	1.3	2.0	2.2
L034	3.5	3.4	3.7	2.3	3.6	1.9	2.9	2.7	2.7	2.4	3.1	4.7	5.3
L035	2.8	1.5	2.3	1.9	2.1	1.2	1.7	1.7	1.6	1.4	1.8	2.4	2.2
L036	3.1	3.2	3.1	1.4	3.1	1.6	2.8	2.5	2.2	2.3	2.5	3.8	3.3
L037	3.6	3.4	3.2	2.0	4.0	2.0	3.5	3.0	3.1	3.3	3.6	4.8	3.3
L038	2.9	3.0	2.6	1.8	2.9	1.7	2.5	2.3	2.0	2.1	2.2	3.4	2.9
L039	2.7	3.1	2.9	2.1	2.8	1.6	2.7	2.8	2.3	2.4	2.6	3.8	3.8
L040	3.1	3.0	3.0	2.5	3.1	1.8	3.0	2.5	2.5	2.6	2.9	3.6	2.9
L041	3.6	3.2	2.8	3.4	3.6	2.0	2.9	3.5	2.4	2.5	2.8	4.7	4.2
L042	3.3	2.9	3.0	2.1	2.8	1.6	2.0	2.4	2.2	1.5	2.4	3.0	2.8
L043	3.4	3.2	3.4	2.9	3.5	1.9	2.7	3.3	3.0	2.0	3.4	4.1	3.6
L044	3.6	3.2	3.5	3.1	3.7	2.0	3.2	3.3	2.6	1.8	2.9	4.6	4.1
L045	3.5	3.2	2.9	2.0	3.5	2.0	2.1	3.1	2.6	1.8	3.0	4.2	3.9
L046	3.6	3.5	3.5	3.3	4.0	2.1	2.6	3.9	3.3	2.3	3.7	4.7	4.0
L047	3.1	3.1	2.5	2.4	3.3	2.1	1.9	3.3	2.5	1.7	2.8	4.6	4.2
L048	3.1	3.4	4.1	2.8	3.6	2.1	2.5	3.4	3.6	2.3	4.1	4.7	4.0
L049	3.4	3.3	3.4	3.2	3.7	2.1	2.1	3.6	3.0	2.0	3.5	5.2	4.5
L050	4.1	4.1	3.0	3.0	4.4	2.6	1.9	4.2	2.8	1.8	3.3	5.9	5.4
L051	5.6	5.6	4.4	5.1	5.3	1.8	4.1	4.2	2.8	3.0	3.1	7.2	5.4
L052	8.6	6.9	4.8	7.1	7.2	1.8	4.1	4.4	2.7	2.7	3.0	6.9	4.5
L053	3.7	4.2	2.8	2.9	3.6	2.0	3.1	3.1	3.0	3.2	3.5	5.3	3.7
L054	3.9	4.3	2.3	4.7	3.9	2.4	2.3	3.0	2.1	2.2	2.4	6.1	4.3
L055	3.0	3.4	1.9	3.6	2.8	1.8	2.1	2.4	2.0	2.2	2.3	4.7	3.2
L056	2.7	3.3	1.8	1.7	2.6	1.3	2.0	1.8	1.9	2.1	2.2	2.7	3.0
L057	3.1	3.7	2.2	2.6	3.0	1.6	2.4	2.7	2.3	2.5	2.8	3.8	5.0
L058	2.4	2.8	0.6	2.1	2.0	2.0	0.8	1.7	1.0	1.0	1.1	2.0	2.3
L059	3.0	3.7	1.7	2.2	2.8	1.4	2.2	2.2	2.4	2.7	2.8	2.8	3.5
L060	3.8	4.0	0.9	2.5	3.1	2.5	1.0	2.5	1.0	1.1	1.2	3.4	4.1
L061	3.1	4.1	2.9	3.5	3.8	0.9	2.8	3.8	2.2	2.3	2.5	4.5	4.7
L062	1.5	1.9	1.3	2.0	2.0	0.6	1.3	1.5	1.1	1.1	1.2	1.3	1.6
L063	2.8	3.9	2.5	3.2	3.5	1.1	2.5	3.1	2.0	2.1	2.2	3.5	4.0
L064	2.5	3.4	3.0	2.9	3.1	1.1	2.5	2.4	3.2	2.8	2.2	2.9	3.3
L065	2.3	3.2	2.9	2.6	3.1	0.9	2.9	3.4	3.0	2.7	2.2	3.5	4.4
L066	2.7	3.3	4.0	2.6	2.9	0.9	3.4	3.6	2.5	2.6	2.8	4.2	4.7
L067	2.3	2.8	2.0	2.3	2.4	0.7	2.1	2.3	1.6	1.7	1.8	2.7	3.0
L068	6.0	7.2	6.7	5.1	5.8	2.2	7.2	5.0	5.5	5.8	5.8	4.8	6.4
L069	2.7	3.4	4.7	3.0	2.8	0.9	5.2	3.1	3.9	4.4	4.6	3.6	4.3
L070	4.2	5.1	3.6	4.6	4.4	1.0	3.2	4.7	2.0	2.2	2.3	6.9	7.8
L071	2.8	3.2	2.8	2.7	2.8	1.8	2.8	2.9	2.3	2.5	2.6	3.1	3.4
L072	2.5	3.1	2.5	2.2	2.5	1.6	2.6	2.9	2.2	2.4	2.5	2.9	3.4
L073	2.5	3.1	2.6	2.1	2.5	1.4	2.3	2.7	2.8	2.3	3.1	2.7	3.1
L074	2.4	3.0	2.6	2.3	2.5	1.4	2.4	2.5	2.9	2.4	3.2	2.0	3.0
L075	2.7	3.2	3.0	2.4	2.7	1.5	2.6	2.4	3.2	2.7	3.6	2.8	3.0
L076	3.3	3.8	4.1	2.3	3.1	2.5	3.2	3.0	3.4	2.8	3.8	3.5	3.9
L077	2.6	2.9	3.1	2.0	2.5	1.3	2.7	2.5	3.3	2.7	3.7	2.5	2.9
L078	2.5	3.0	2.0	2.6	2.5	1.8	1.9	2.8	1.5	1.7	1.7	2.9	3.7
L079	2.6	2.9	2.2	1.8	2.5	1.5	2.2	2.4	1.9	2.0	2.1	2.9	3.1
L080	3.6	3.7	3.7	2.6	3.6	2.5	3.4	4.1	2.2	2.4	2.5	4.6	5.4
L081	4.6	5.9	3.5	4.6	4.4	2.6	3.0	5.1	2.1	2.1	2.4	7.3	7.1
L082	3.6	4.1	2.7	3.1	3.4	2.3	2.5	3.7	2.1	2.1	2.4	5.0	4.3
L083	4.9	6.1	3.1	5.3	5.0	2.6	3.6	6.2	2.0	2.3	2.3	10.2	10.6
L084	3.5	4.6	2.5	4.0	3.3	2.3	2.7	4.1	1.6	1.8	1.8	6.5	6.1
L085	3.0	3.8	2.0	2.4	2.6	2.0	2.4	2.6	1.6	1.7	1.7	3.2	1.8
L086	3.0	3.6	1.3	2.8	2.6	2.5	2.0	2.6	1.5	1.6	1.6	3.7	3.5
L087	3.0	3.5	1.1	2.9	2.7	2.3	1.9	2.7	1.2	1.3	1.4	3.4	2.3
L088	3.0	3.6	1.2	3.9	2.8	1.5	1.7	2.4	1.0	1.1	1.1	3.8	3.6
L089	3.0	3.6	3.1	2.7	2.6	1.7	2.9	2.4	2.0	2.3	2.4	3.9	3.9
L090	2.9	3.4	2.4	2.4	2.4	1.9	2.3	2.5	1.7	1.9	1.9	2.9	3.0
L091	5.7	5.2	5.3	6.2	5.3	2.7	5.0	9.1	2.8	3.2	3.2	8.5	7.7
L092	3.1	4.0	2.2	2.9	3.0	1.9	2.3	3.1	1.8	2.0	2.1	4.7	4.3
L093	3.5	4.3	2.5	3.0	3.3	1.9	2.4	3.7	1.7	1.9	2.0	5.2	5.1
L094	2.8	3.7	2.1	2.9	2.7	2.0	2.2	2.8	1.8	1.9	2.0	3.8	3.1
L095	3.2	3.7	2.8	2.2	3.0	2.5	3.2	2.5	2.9	3.1	3.3	4.0	2.2
L096	5.6	4.8	3.0	5.5	5.3	2.7	3.2	7.6	3.3	3.5	3.4	8.5	7.5
L097	2.8	3.4	2.3	2.2	2.6	2.2	2.4	2.0	2.1	2.2	2.3	3.3	1.8
L098	3.5	4.0	2.5	2.7	3.4	2.4	3.0	3.1	3.0	3.3	3.4	4.6	2.6
L099	2.9	3.3	2.1	2.2	2.4	2.2	2.3	1.9	1.9	2.1	2.1	2.8	1.5
L100	3.2	3.6	2.3	2.2	2.9	2.2	2.5	2.1	2.1	2.3	2.4	3.4	2.1
L101	2.8	3.3	1.9	2.2	2.3	2.0	2.0	1.9	1.4	1.6	1.6	3.3	2.7
L102	3.5	4.3	2.5	2.6	3.4	2.1	2.6	2.8	1.9	2.0	2.1	5.2	5.5
L103	3.3	3.8	2.4	2.8	3.1	2.0	2.5	2.4	2.0	2.2	2.3	3.1	2.0
L104	2.9	3.6	2.2	2.1	2.8	2.2	2.5	2.2	2.1	2.3	2.3	3.6	2.1
L105	2.7	3.0	2.1	2.2	2.6	2.0	2.3	2.0	1.8	2.0	2.0	3.1	2.6
L106	2.8	2.9	2.1	1.7	2.7	2.1	2.4	1.0	2.0	2.2	2.3	3.1	1.8
L107	2.9	3.5	2.2	2.1	2.8	2.1	2.4	2.2	2.0	2.2	2.3	3.2	2.5
L108	2.7	3.1	1.8	2.2	2.4	2.1	1.9	1.9	1.5	1.6	1.7	2.8	2.6
L109	2.5	3.1	1.9	1.9	2.3	2.0	2.0	1.7	1.5	1.7	1.7	2.6	2.5
L110	2.7	2.7	2.1	2.6	2.1	1.7	2.3	2.4	1.8	2.0	2.1	2.9	2.4
L111	2.9	2.0	1.8	1.9	2.3	1.1	2.0	2.0	1.5	1.6	1.7	2.3	2.2
L112	2.6	2.4	1.9	2.1	2.1	1.0	2.1	1.8	1.6	1.8	1.8	2.3	2.2
L113	2.7	2.6	1.8	1.8	2.2	1.7	2.0	1.9	1.6	1.7	1.7	2.3	2.1
N	113	113	113	113	113	113	113	113	113	113	113	113	113
Min	1.4	1.5	0.6	1.4	1.3	0.6	0.8	0.3	0.5	0.5	0.7	1.0	1.0
Max	16.7	12.5	15.2	11.5	19.7	3.7	12.9	15.2	12.4	8.2	14.5	15.4	13.5
Mean	3.8	4.0	3.4	3.4	3.9	2.0	3.2	3.7	3.0	2.7	3.3	5.1	4.7
Standard error	0.2	0.1	0.2	0.2	0.2	0.1	0.2	0.2	0.2	0.1	0.2	0.3	0.2
Variance	4.2	2.3	4.6	3.3	5.1	0.3	3.0	4.1	3.2	1.7	4.3	7.2	6.3
Standard deviation	2.1	1.5	2.1	1.8	2.3	0.6	1.7	2.0	1.8	1.3	2.1	2.7	2.5
Median	3.3	3.7	2.9	2.7	3.3	2.0	2.7	3.1	2.5	2.3	2.8	4.2	4.0
25 percentile	2.8	3.2	2.2	2.2	2.6	1.6	2.2	2.4	1.9	1.9	2.1	3.1	2.9
75 percentile	4.1	4.6	3.8	3.8	5.0	2.3	3.6	4.5	3.3	3.3	3.8	6.9	6.1
Coefficient of variation	53.9	37.6	62.4	54.0	57.4	29.0	54.6	55.6	60.5	47.4	62.1	52.9	53.8

## 2 Experimental design, materials and methods

### 2.1 Study area and sampling procedure

**Fig. 5:**
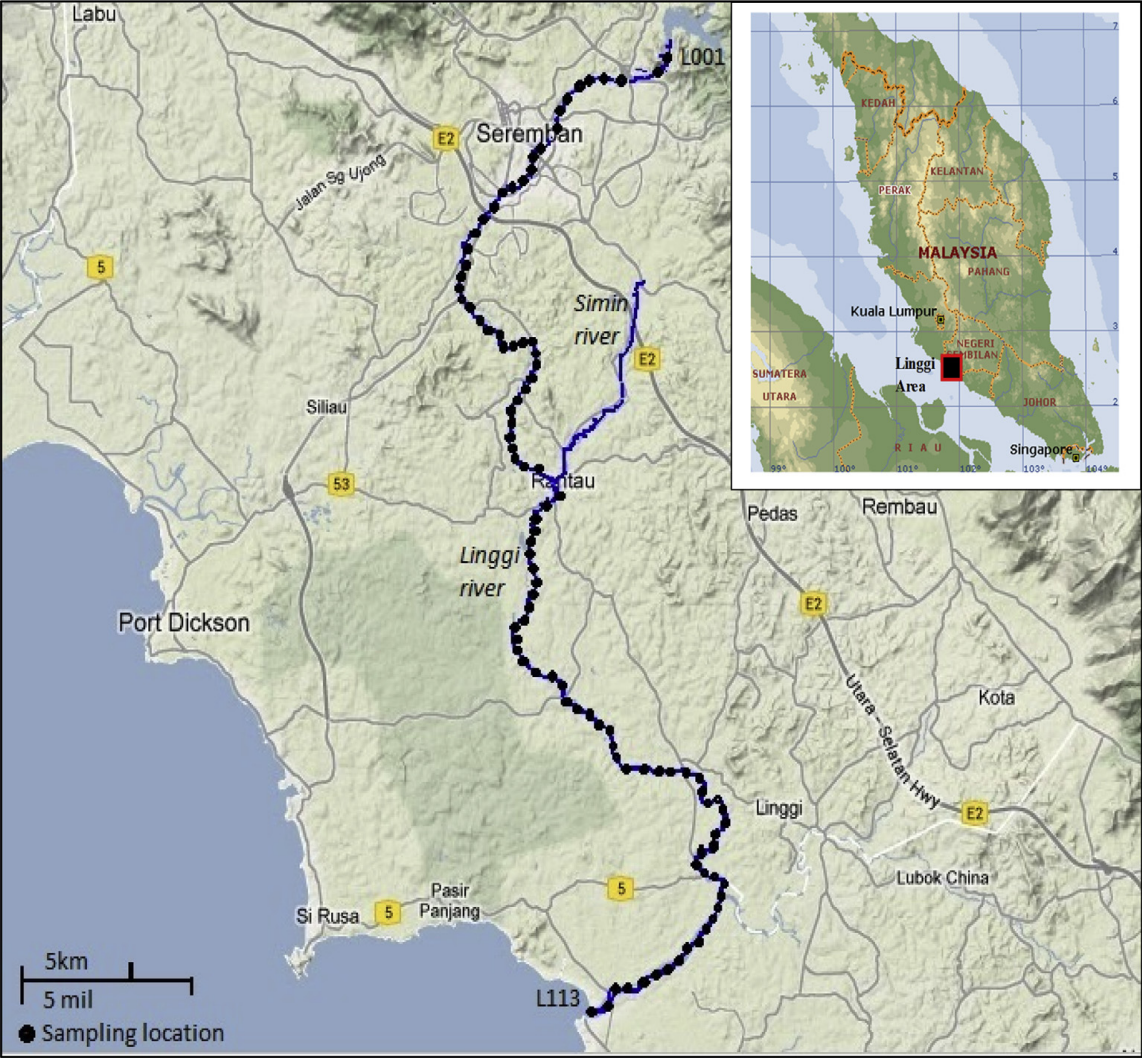
Map showing the sampling locations along Linggi River.

A total of 113 sampling locations of sediment samples were selected along the Linggi River (Fig. 5) located between latitude 2° 22′24.77″ N to 2° 53′55.24″ N and longitude 102° 06′49.46″ E to 102° 12′48.37″ E. Sediment samples were collected using grab sampler and transferred into pre-cleaned polyethylene bottles and transported to the laboratory. A portion of sediment samples was kept for particle size analysis and the remaining sediment samples were dried in an oven at 60 °C until constant weight. The dried sediments were ground using agate mortar to achieve a homogenous powder form prior to analysis by neutron activation analysis (NAA) technique and inductively coupled plasma – mass spectrometer (ICP-MS).

### 2.2 Particle size analysis

Approximately 0.5 g of the sediment sample was mixed with double distilled water and followed by addition of 1 mL of sodium hexametaphosphate as an agent to avoid agglomeration. The sediment samples underwent ultrasonic process for 30 seconds prior to analysis. Analysis of particle size were done by using Honeywell Microtrac (model ×100) equipped with laser diffraction capable of measuring sizes ranging from 0.02 to 700 μm to determine the average and distribution of particle size.

### 2.3 Neutron activation analysis (NAA) technique

Approximately 200 mg of sediment samples, SRM (IAEA SL-1), IAEA Soil-7 and blank were simultaneous irradiated in the PUSPATI TRIGA MARK II Reactor, Malaysian Nuclear Agency for 6 hours at 750 kW power and with a thermal flux of 4.0 × 10^12^ n.cm^−2^.s^−1^. The counting of the sediment sample, blank, SRM (IAEA SL-1), IAEA Soil −7 (as comparator) was done by gamma spectrometer. Calculation of REEs concentration was performed according to procedure as described by Elias et al., 2018 [11].

### 2.4 Inductively couple plasma – mass spectrometer (ICP-MS) analytical method

Approximately 200 mg of homogenised powder sediment sample and SRM (BHVO-1) were digested using a hot block digester (model Vision). The replicate sediment samples and SRM were digested using a mixture of HNO_3_, H_2_O_2_, and HF. The SRM (BHVO-1) was used as quality assurance and quality control in the analytical method analysis. The SRM (BHVO-1) measurement followed the same procedure as a sample analysis. The reagent blank acid used in digestion process was monitored throughout the analysis and used to correct the analytical results. The method for digestion process of the sediment samples and SRM (BHVO-1) was described by Elias et al., 2018 [12]. The isotopes of ^141^Pr, ^158^Gd, ^165^Ho, ^166^Er, and ^169^Tm were measured by using ICP-MS (Perkin Elmer model ELAN 6000).

### 2.5 Enrichment factor (EF) of Linggi sediment

The Linggi river sediments were normalised to mud from Queensland (MUQ). MUQ was used as a reference shale value. Enrichment factor (EF) of Linggi sediment was calculated according to equation (1) to evaluate the enrichment of Linggi River sediment. Enrichment of the Linggi River sediment was then categorised according to the following: no enrichment (background shale), minor, moderate, moderately severe, severe, very severe and extreme enrichment.
